# Predictive classification and regression models for bioimpedance vector analysis: Insights from a southern Cuban cohort

**DOI:** 10.2478/joeb-2025-0012

**Published:** 2025-08-04

**Authors:** Jose Luis García Bello, Taira Batista Luna, My Phuong Pham-Ho, Minh Tho Nguyen, Alcibíades Lara Lafargue, Héctor Manuel Camué Ciria, Yohandys A. Zulueta

**Affiliations:** Autonomous University of Santo Domingo (UASD), San Francisco de Macorís Campus, Dominican Republic; Autonomous University of Santo Domingo (UASD), UASD Nagua Center, Dominican Republic; Faculty of Chemical Engineering, Ho Chi Minh City University of Technology (HCMUT), District 10, Ho Chi Minh City, 70000 Vietnam; Vietnam National University Ho Chi Minh City, Linh Trung, Thu Duc, Ho Chi Minh City, 70000 Vietnam; Lab. for Chem. Comput. and Modelling, Institute for Comput. Science and Art. Intell., Van Lang University, Ho Chi Minh City, 70000 Vietnam; Faculty of Applied Technology, School of Technology, Van Lang University, Ho Chi Minh City, 70000 Vietnam; National Center for Applied Electromagnetism (CNEA), Universidad de Oriente CP 90500, Santiago de Cuba, Cuba; Departamento de Física, Facultad de Ciencias Naturales y Exactas, Universidad de Oriente, CP 90500, Santiago de Cuba, Cuba

**Keywords:** BIA, BIVA, characteristic frequency, characteristic reactance, characteristic resistance

## Abstract

This study used predictive models to explore the link between bioparameters at characteristic frequency and their positions within tolerance ellipses in a southern Cuban cohort. The database includes 367 individuals (235 females, 132 males) aged 18–86. Among them, 61 had cancer, while 306 were healthy. After balancing the data, the analysis used 16 bioimpedance-based characteristics along with other anthropometric and location factors. The results showed that characteristic frequency bioparameters (Zc, θc, Xcc, and Rc) are key for assessing health and location. There was a strong agreement between experimental and predicted values for Zc, θc, Xcc, and Rc across various categories. Cancer patients showed higher Zc and slightly lower *θ*_*c*_ and Xcc values, attributed to unbalanced body composition and cell membrane deterioration. Females exhibited higher Zc and Xcc values, indicating better cell membrane integrity. Predictions are consistent across quartiles and percentiles, with lower *θ*_*c*_ observed in higher quartiles and centiles where more cancer patients are located. Variations in Rc values across different BIVA statuses demonstrated the model's robustness in estimating impedance parameters in diverse physiological conditions. These predictive models are significant for assigning locations without developing BIVA methods, enhancing clinical assessments and health monitoring.

## Introduction

Bioimpedance technology presents a versatile and noninvasive method for cancer detection and monitoring. Its use in detecting and tracking mammary, neck, skin, and breast cancers showcases its potential to enhance early diagnosis, monitor treatment efficacy, and improve patient outcomes. As research advances, bioimpedance could become a crucial component of personalized cancer care, equipping clinicians with essential tools for more accurate diagnosis and effective management [1,2,3,4,5,6,7,8,9,10,11,12].

Bioimpedance technologies, which measure the resistance and reactance of biological tissues to an applied electrical current, have demonstrated significant potential in oncology [1,2,3,4,5,6,7,8,9,10,11,12,13]. This non-invasive method offers valuable insights into tissue composition and physiological changes, making it a promising tool for cancer detection and monitoring [1,2,3]. For instance, mammary cancer, commonly known as breast cancer, is one of the most prevalent cancers among women [13,14,15,16]. Early detection and accurate monitoring are essential for improving patient outcomes [17,18,19]. Recent studies have explored the application of bioimpedance in various types of cancer, including mammary, neck, skin, and breast cancers [13,14,15,16].

Bioimpedance techniques, including single-frequency (SF-BIA) and multi-frequency (MF-BIA) bioimpedance, are used to measure physiological parameters. SF-BIA, which utilizes a single frequency (50 kHz) of electrical current, is a simple and cost-effective method often employed in healthcare and fitness settings [20,21,22,23,24]. However, it may not accurately measure extracellular and intracellular water content due to varying electrical properties of tissues and fluids at different frequencies [20,21,22,23,24]. MF-BIA and impedance spectroscopy (SBIA) uses multiple frequencies disclosing more detailed information on the cellular and molecular properties of tissues and fluids [20,21,22,23,24]. Another bioimpedance method is the BIA vector analysis (BIVA). BIVA is a non-invasive method that evaluates body composition and hydration status by measuring electrical impedance at a low frequency. The BIVA method standardizes Bioelectrical Impedance Analysis (BIA) measurements by height and represents them as bivariate vectors with confidence intervals, depicted as ellipses on the R-Xc plane. This technique's key advantage is its ability to provide simultaneous information about changes in tissue hydration and soft-tissue mass, independent of regression equations or body weight. BIVA has been extensively utilized to study hydration across various diseases [20,21,22,23,24,25,26,27,28] and to conduct general body composition assessments in patients with lung cancer [27, 29] and head and neck cancers [25,26,27,28].

Studies have demonstrated that BIVA can effectively differentiate between cancer patients and healthy individuals by analysing parameters such as impedance, phase angle, and reactance [25,26,27,28]. For instance, cancer patients often exhibit lower phase angles and higher impedance values, indicating altered cellular integrity and body composition due to the disease. In addition, BIVA has been utilized to assess hydration status in advanced cancer patients, revealing significant associations between hydration levels and clinical outcomes. Research has shown that lower hydration status is linked to increased symptom intensity and shorter survival times in cancer patients [25,29,30]. The integration of BIVA into routine assessments highlights its potential as a valuable tool in oncology and general health monitoring [25,29,30].

In a previous work, the integration of various bioimpedance modalities was used to evaluate the health status of southern Cuban populations. We demonstrated that this integrated methodology serves as a sensitive complementary tool, adept at distinguishing between healthy individuals and cancer patients. In addition, it confirms that the phase angle value at the characteristic frequency may be a robust indicator of overall health status of individuals, similar to the reported at 50 kHz [31].

Determining the location of individuals within the tolerance ellipses derived by BIVA is crucial for accurately assessing their health and nutritional status [25,26,27,28,29,30,31]. Location variables contemplate the BIVA status, quartile and centile. These ellipses provide a graphical representation of an individual's impedance vector relative to a reference population [26,27,28,29]. By pinpointing where an impedance vector falls within these ellipses, clinicians can gain valuable insights into their body composition, hydration levels, and cellular health [26,27,28,29]. For example, individuals positioned within the upper regions of the ellipses typically exhibit better cell membrane integrity and hydration status, while those in the lower regions may indicate dehydration or cellular function [25,26,27,28,29,30]. Moreover, the location within the tolerance ellipses can help identify early signs of malnutrition, overhydration, or other health issues that may not be immediately apparent through conventional assessments [26,27,28,29,30]. This proactive approach is particularly beneficial for managing chronic conditions, such as heart failure, renal disease, and cancer. The ability to determine an individual's location within the BIVA tolerance ellipses enhances the precision and effectiveness of health assessments, leading to better-informed clinical decisions and improved patient outcomes [25,26,27,28,29,30,31].

Interest in artificial intelligence (AI) has grown significantly in recent years, driven by continuous advancements in computer science. AI has proven particularly useful in data management and various scientific fields, including medicine [32,33,34,35,36]. One area where AI holds great potential is diagnostic imaging, as it can aid in identifying various conditions, including body composition [32,33]. This capability may further contribute to predicting health outcomes across different diseases [32,36]. For instance, a combination of bioimpedance measurements and machine learning analysis was conducted in an infant-juvenile cohort from the eastern Cuban region [34,35]. The classification model demonstrated that, aside from body mass index, alternative indicators can serve as predictors of weight status [34,35]. Additionally, the regression learner model accurately predicted the weight status of the subjects with high precision [34,35].

Recently, a cancer predictive model was developed using bioimpedance measurements and machine learning methods [36]. The results revealed that the classification model identified two robust parameters for predicting health status: impedance, total body water, and phase angle, which showed significant relevance [36]. The phase angle predictions align with previous reports on other pathologies, indicating that higher phase angle values are associated with better health status. Furthermore, males tend to have higher phase angle values compared to females [36].

To our knowledge, there is no reports combining machine learning with BIVA studies for prediction of location classes. In the present work, we employed predictive classification and regression learner models to investigate the association between bioparameters of subjects, derived at the characteristic frequency, and their locations within tolerance ellipses for a cohort from the southern Cuban region. The model developed in this study plays a crucial role in location assignment without relying on BIVA methods, improving clinical evaluations and health monitoring.

## Materials and Methods

### Population recruitment and ethical approval

A descriptive, retrospective, randomized study was conducted on patients who visited the “Conrado Benítez” Teaching Oncology Hospital in Santiago de Cuba for suspected cancer during the periods of March–April and July–August 2002. The hospital was chosen for its suitable facilities and accommodations, despite participants hailing from various locations across eastern Cuba. The study comprised 367 subjects (235 females and 132 males) aged 18 to 86 years. Among them, 61 were diagnosed with different types of cancer pathologically, while 306 were healthy. Participants were recruited following strict ethical guidelines and medical practices as outlined by the Health General Law of the Ministry of Public Health of the Republic of Cuba (Number 41, July 13, 1983, updated in 2010).

Following the Helsinki Declaration, the research was approved by the ethics committees and scientific councils of the Oncological Hospital “Conrado Benítez.” Additionally, data on bioelectrical parameters of cancer patients and healthy individuals were retrieved from a database archived at the National Center for Applied Electromagnetism (CNEA); (ISBN: 978-959-207-679-2). The diagnosis of the patients was made through pathological anatomy. The samples were taken using the fine needle aspiration cytology technique. Anatomical pathology laboratory diagnoses revealed that female patients had various types of cancer with different stages, including breast and cervical cancer, whereas male patients were diagnosed with more aggressive cancers, such as skin melanoma, colon cancer, lung neoplasm. Further details can be found in Ref [11].

### Informed consent

Informed consent has been obtained from all individuals included in this study.

### Ethical approval

The research related to human use has been complied with all relevant national regulations, institutional policies and in accordance with the tenets of the Helsinki Declaration, and has been approved by the authors' institutional review board or equivalent committee.

### Machine learning models

Achieving balanced data is essential for successful machine learning studies [32,33,34,35,36,37,38,39,40,41]. In our study, we ensured data balance by implementing random oversampling for the minority class (cancer) and random undersampling for the majority class (healthy). The final dataset included 621 individuals, aged between 18 and 86 years (382 females and 239 males), with 316 diagnosed with cancer and 306 healthy participants. To prevent overfitting, a cross-validation technique is used, in which 95% of the data is designated for training and the remaining 5% is reserved for validation. This approach ensures that the model generalizes well to unseen data rather than memorizing patterns specific to the training set [37,38,39,40,41].

For the machine learning study, the features include: health status (cancer, healthy), sex, high frequency resistance (R_inf_), characteristic frequency (*f*_c_), Cole parameter (α), corrected resistance (r_c_), capacitive reactance Xc, height, weight, age, resistance (Rc), capacitive reactance (Xcc), impedance absolute value $Z_{c}=\sqrt{Rc^{2}+Xcc^{2}}$ and phase angle (*θ_c_*) at the characteristic frequency and location variables (BIVA status, quartile and centile). For the classification models, the response are the location features and the health status, while for the regression, the bioelectrical parameters at the characteristic frequency (Rc, Xcc, *θ_c_* and *Z_c_*). In this sense, one can explore the role of the anthropometric and bioelectric parameters for location assignments at the tolerance ellipses, usually made by bioimpedance vector analysis.

Several metrics can be used to assess the accuracy of machine learning models [39,40,41]. Metrics for classification models are derived from the confusion matrix [39,40,41]. For classification models, the accuracy is defined as:

(1) $$\mathrm{Accuracy}=\frac{\mathrm{TP}+\mathrm{TN}}{\mathrm{TP}+\mathrm{TN}+\mathrm{FP}+\mathrm{FN}}$$

where (TP ) is true positives, (TN) is true negatives, (FP) is false positives, and (FN) is false negatives.

The precision, which indicates the amount of the predicted positive instances that are actually positive is defined as follows [39,40]:

(2) $$\mathrm{Precision}=\frac{\mathrm{TP}}{\mathrm{TP}+\mathrm{FP}}$$

The recall describes how well the model captures all the positive instances [39,40]:

(3) $$\mathrm{Recall}=\frac{\mathrm{TP}}{\mathrm{TP}+\mathrm{FN}}$$

F1-Score is defined as [39,40]:

(4) $$F1-\mathrm{Score}=2\times \frac{\mathrm{Precision}\times \mathrm{Recall}}{\mathrm{Precision}+\mathrm{Recall}}$$

which estimate the balance between Precision and Recall.

In addition, for regression models the common metrics are: the coefficient of determination (*R*^2^), mean absolute error (MAE), root mean square error (MSE) and its root mean square error (RMSE) defined by equations (5) [39,40]:

(5) $$\begin{array}{l} R^{2}=1-\frac{\sum_{i=1}^{n}{\left(y_{i}-{\hat{y}}_{i}\right)}^{2}}{\sum_{i=1}^{n}{\left(y_{i}-{\bar{y}}_{i}\right)}^{2}}\\ \text{MAE}=\frac{1}{n}\sum_{i=1}^{n}\left|y_{i}-{\hat{y}}_{i}\right|\\ \text{MSE}=\frac{1}{n}\sum_{i=1}^{n}{\left(y_{i}-{\hat{y}}_{i}\right)}^{2}\\ \text{RMSE}=\sqrt{\mathrm{MSE}} \end{array}$$

where *n* represents the number of elements in the database, *y*_*i*_ the observed value of the dependent variable, *ŷ*_*i*_ the predicted value of the dependent variable, and *ȳ*_*i*_ the mean value of the observations. When *R*^2^ ≅ 1 the model tends to be perfect, analogously for MAE, MSE and RMSE values [39,40].

### Bioimpedance Measurements

Bioimpedance parameters were measured using a BioScan 98^®^ model bioimpedance analyzer (Biológica Tecnología Médica S.L., Barcelona, Spain) with a tetrapolar whole-body configuration. Participants, who fasted for at least 3 hours, emptied their bladders, and refrained from exercise and alcohol for 12 hours prior, were included in the study. MF-BIA measurements were taken at frequencies ranging from 10 to 250 kHz using Ag/AgCl electrodes model 3M Red Dot 2560 (3M, Ontario, Canada). The study was conducted in a room maintained at 23°C with 60–65% relative humidity. Volunteers lay on a non-conductive surface without clothing or pillows, with their arms positioned 30° away from the chest and legs spread 45° apart. The injector electrodes were placed (after cleaning the skin with 70% alcohol) on the inner side of the dorsal surfaces of the hands and feet, near the metatarsophalangeal and third metacarpal joints. The detector electrodes were positioned between the distal ends of the ulna and radius at the pisiform prominence and at the midpoint between both malleoli. A 5 cm gap was maintained between detector and injector electrodes during measurements.

## Results and Discussion

### Estimation of subject location on the tolerance ellipse with the aid of classification models

With the balanced database, we perform classification models to predict the location features. Table 1 collects the best model and the metrics describing the model performance and generality of each location feature. The Fine Tree model is the most effective in describing health and BIVA statuses, with accuracies of 92.80% and 99.50%, respectively. Meanwhile, the Linear Support Vector Machine and Random under-sampling Boosted Tree approach (RUSBoosted Tree) are the top models for describing quartile and centile responses, achieving accuracies of 100% and 97%, respectively.

**Table 1:** Response models and their respective metrics for the classification of health status and location variables. The column Class includes Health Status (Cancer, Healthy), Quartile (1, 2, 3, 4), Centile (50, 75, 95, and 100%), and BIVA status (11, 12, 13, 14, 21, 22, 23, 31, 32, 33, 41, 42, 43, 44).

**Response**	**Model**	**Accuracy**	**Class**	**Precision**	**Recall**	**F1-score**
Health status	Fine Tree	92.80%	Cancer	0.912	0.951	0.931
			Healthy	0.947	0.905	0.926
BIVA status	Fine Tree	99.50%	11	1.000	1.000	1.000
			12	1.000	1.000	1.000
			13	1.000	1.000	1.000
			14	1.000	1.000	1.000
			21	1.000	1.000	1.000
			22	1.000	1.000	1.000
			23	0.857	0.750	0.800
			31	1.000	1.000	1.000
			32	1.000	1.000	1.000
			33	0.889	0.941	0.914
			41	1.000	1.000	1.000
			42	1.000	1.000	1.000
			43	1.000	1.000	1.000
			44	1.000	1.000	1.000
Quartile	Linear SVM	100%	1	1.000	1.000	1.000
			2	1.000	0.983	0.991
			3	0.990	1.000	0.995
			4	1.000	1.000	1.000
Centile	RUS Boosted Tree	97%	50%	0.988	0.984	0.986
			75%	0.959	0.953	0.956
			95%	0.948	0.938	0.943
			100%	0.957	1.000	0.978

**Fig. 1:**
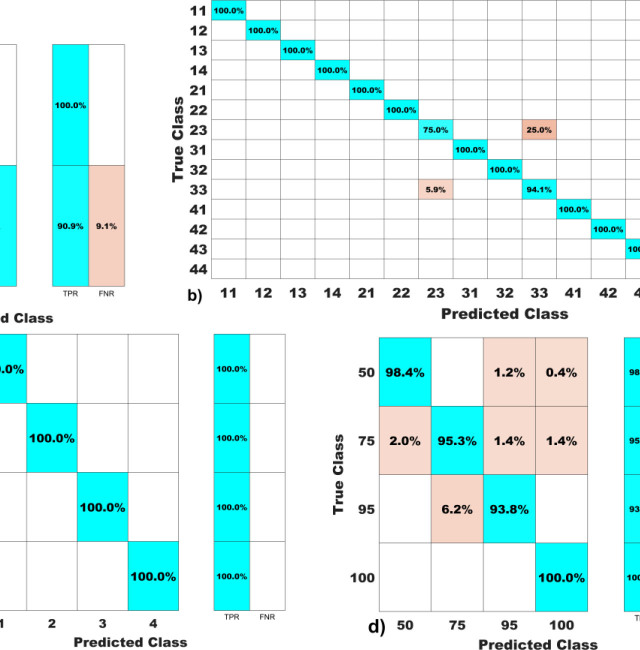
Confusion matrix of trained models: a) Health status, b) BIVA status, c) quartile and d) centile responses.

The confusion matrices presented in Figure 1 illustrate the model's performance across various classification tasks. For health status (Figure 1a), the matrix reveals a high true positive rate (TPR) for healthy individuals, with 96.9% correctly identified and only 3.1% misclassified. The model shows excellent accuracy in distinguishing between healthy individuals and those with health issues, demonstrated by the 100% correct classification of the cancer (C) class. For BIVA status (Figure 1b), which encompasses 44 classes, most achieved 100% classification accuracy. Minor misclassifications were observed between classes 33 and 34, with a small percentage (4.5%) incorrectly classified.

The overall TPR of 99.5% highlights the model's high precision in identifying BIVA statuses correctly. The Quadrant Responses matrix (Figure 1c) displays perfect classification, achieving 100% accuracy across all four classes, indicating an exceptional ability to differentiate quadrant responses without any errors. For Centile Responses (Figure 1d), the matrix shows slightly lower but still high performance, with true classes 50%, 75%, and 95% having some minor misclassifications. Notably, class 100% is perfectly classified. The overall TPR of 95.3% demonstrates robust model performance, though there is some room for improvement in distinguishing closely related centiles. Collectively, these matrices underscore the models' effectiveness in accurately classifying various health statuses, BIVA parameters, quadrant responses, and centile categories, with high true positive rates and minimal misclassifications.

Feature importance analysis using a Pareto chart is a powerful method for identifying and visualizing the most significant predictors in a classification model [37,38,39]. By ranking features according to their importance scores and displaying them in a Pareto chart, one can easily discern the few key features that contribute most to the model's predictive power [37,38,39]. Applied to feature importance, this means that a small number of features typically account for the majority of the model's performance. In the Pareto chart, features are ordered from highest to lowest importance, with a cumulative line illustrating their collective contribution. This visual representation aids in prioritizing features for model improvement, simplifying complex datasets, and focusing efforts on the most impactful predictors [37,38,39].

**Fig. 2:**
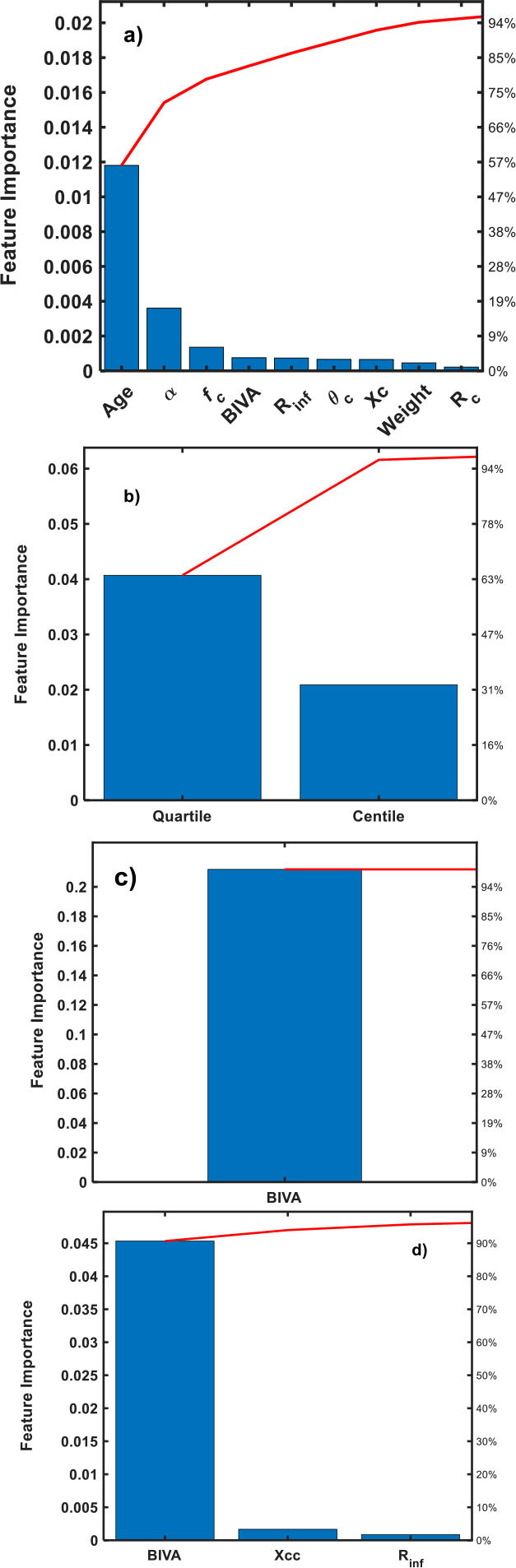
Feature importance of trained models: a) Health status, b) BIVA Status, c) quartile and d) centile responses.

Figure 2 displays the feature importance of each response. For health status model (Figure 2a), it can be note that the most influencing feature is the age with a 95% of importance score, followed by α, fc, BIVA status (BIVA), R_inf_, *θ*_*c*_, Xc, weight and Rc, in descendent order. These findings align with the natural progression of life, influenced by biological changes, environmental factors, and lifestyle habits that accumulate over time. Cole parameter (α) and fc with a ~18% and ~8% of importance, respectively, also align with life evolution. Both parameters undergo logical variations in consequence of biological changes. In the case of BIVA status response (Figure 2b), there are two top ranking features: quartile (~97%) and centile (~31%). The quartile (Figure 2c) is highly influenced by the BIVA status (~96%) and centile response (Figure 2d) by the BIVA (~96%), Xcc (~5%) and R_inf_ (~2%) bioparameters. These finding align well with the BIA vector analysis location in the tolerance ellipse, where the quartile determine the body composition and hydration status and the centile offers insights into cell integrity, muscle mass and fluid balance [25,26,27,28,29,30,31].

The phase angle is a well-established marker for assessing cell membrane health and integrity, bearing significant implications for cancer prognosis [41,42,43,44]. A comprehensive study with 2625 participants demonstrated a positive and substantial correlation between phase angle and cancer survival rates. Specifically, patients exhibiting low phase angle values were found to be 23% less likely to survive compared to those with higher values [41]. Additionally, phase angle at the time of diagnosis is recognized as a crucial prognostic factor for survival among patients with advanced head and neck cancer [42]. Previous research has indicated that phase angle shows a moderate to strong correlation with body composition and physical function, while its correlation with nutritional status, complications, survival, quality of life, and symptoms tends to be weaker. These findings underscore the utility of phase angle as a robust indicator in clinical assessments, particularly in the context of cancer prognosis [43,44].

In addition, phase angle, Xc and R determine the location in the tolerance elipses in BIVA studies [25,26,27]. By analysing the position of an individual's impedance vector within these ellipses, researchers can assess hydration status, cell membrane integrity, and overall cellular health.

Previous reports used single-frequency bioimpedance for BIVA studies. In our previous work, we demonstrated the use of impedance spectroscopy combined with BIVA for accessing the health status by constructing the tolerance elipses at the characteristic frequency instead of the common 50 kHz [31]. In the present study, we found that the bioparameters derived at the characteristic frequency play a crucial role determining the health status and the location parameters.

### The role of bioelectrical parameters at the characteritic frequency estimating the location on the tolerance elipse

**Fig. 3:**
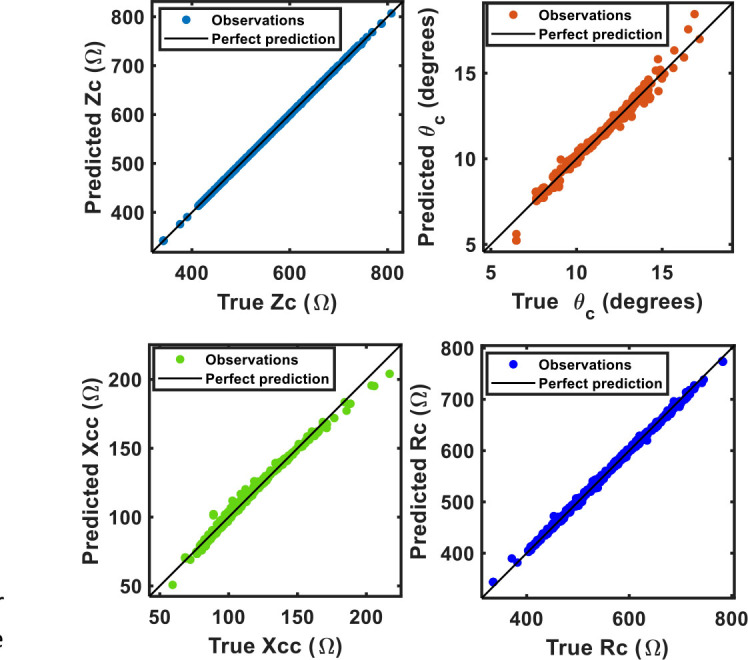
Response vs predicted plot of the selected responses.

This section focuses on developing regression models to predict Zc, Rc, Xcc, and *θ*_*c*_ in order to assess their robustness in determining the location on the tolerance elipses. Table 2 collects the results of the model for each response and their respective accuracy values. The response vs. predicted plots in Figure 3 provides a visual assessment of the models' performance in predicting Zc, Rc, Xcc, and *θ*_*c*_. For Zc, the top-left plot indicates that the model predictions are closely aligned with the actual values, as most data points fall near the line of perfect prediction. This high level of accuracy suggests that the model is highly reliable in predicting impedance values. The characteristic phase angle (*θ*_*c*_), shows a similarly strong agreement between predicted and actual values. The close alignment of data points with the line of perfect prediction underscores the model's effectiveness in estimating phase angle, a critical indicator of cell membrane health and integrity [25,26,27,28,29,30]. In the bottom-left plot, which compares true and predicted values for reactance Xcc, the data points again closely follow the line of perfect prediction. This suggests that the model is adept at estimating reactance, an important measure for assessing cellular health and hydration status [25,26,27,28,29,30,31]. In the case of the characteristic resistance (Rc), shows that the model's predictions are also highly accurate. The data points close alignment with the perfect prediction line indicates that the model can reliably predict resistance values, essential for evaluating total body water and extracellular water content [25,26,27,28,29,30].

**Table 2:** Accuracy parameters of each model.

**Response**	**Model**	**R^2^**	**RMSE**	**MSE**	**MAE**
Zc (Ω)	Linear	1.000	0.351(Ω^2^)	0.123 (Ω)	0.233 (Ω)
*θ*_*c*_ (°)	Linear	0.980	0.237 (°^2^)	0.056 (°)	0.166 (°)
Xcc (Ω)	Linear SVM	0.990	2.378 (Ω^2^)	5.652 (Ω)	1.674 (Ω)
Rc (Ω)	Linear SVM	1.000	4.216 (Ω^2^)	17.773 (Ω)	3.223 (Ω)

The accuracy parameters listed in Table 2 further corroborate the visual findings from Figure 3. The Fine Tree model for health and BIVA statuses demonstrates an R^2^ of 1.00 for characteristic impedance and resistance, indicating perfect prediction. The RMSE, MSE, and MAE values are very low for these models, further emphasizing their high accuracy. The phase angle has an R^2^ = 0.98, with similarly low RMSE, MSE, and MAE values, highlighting its robust prediction accuracy.

For the characteristic reactance, the Linear SVM model shows an R^2^ = 0.99, indicating slightly lower but still high accuracy. The RMSE, MSE, and MAE values reflect this slight decrease in precision but still demonstrate the strong performance.

**Fig. 4:**
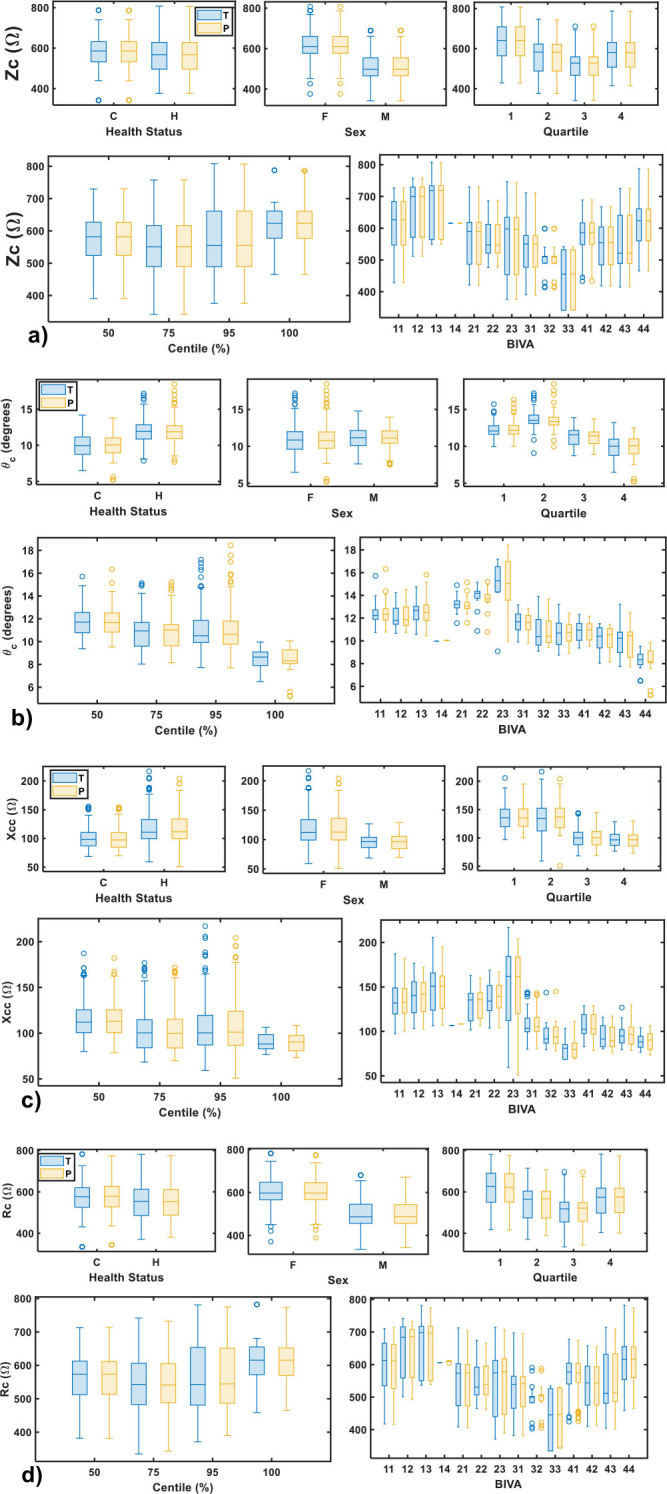
observable and predictions of Zc, *θ*_*c*_, Xcc and Rc across various categories: Health Status (Cancer, Healthy), Sex, Quartile (1, 2, 3, 4), Centile (50, 75,95 and 100%), and BIVA status (11, 12, 13, 14, 21, 22, 23, 31, 32, 33, 41, 42, 43, 44).

Figure 4 displays the observable and predictions of Zc, *θ*_*c*_, Xcc and Rc across various categories: Health Status (Cancer, Healthy), Sex, Quartile (1, 2, 3, 4), Centile (50, 75,95 and 100%), and BIVA status (11, 12, 13, 14, 21, 22, 23, 31, 32, 33, 41, 42, 43, 44).

The box plots in Figure 4 illustrate the close alignment between the experimental and predicted values of Zc, *θ*_*c*_, Xcc and Rc. The blue box plots represent experimental data, while the yellow box plots depict the predicted values. From Figure 4, a strong agreement is observed between the experimental and predicted values across all categories, suggesting that the model effectively captures the characteristic electrical parameters, with high accuracy. For instance, the median value of Zc of observable cancer patients is 585.9 Ω and 567.3 Ω for healthy individuals, and the predicted values are 585.8 Ω and 567.6 Ω for cancer and healthy subjects (Figure 4a). It can be attributed that cancer patients have more unbalanced body composition due to the proper evolution of the pathology [25,29,30]. By sex, female have higher median values of 610.5 Ω as compared with male individuals 497.2 Ω with similar predicted values. These findings can be attributed to the fact that woman accumulates more adipose tissues than man in general. Concerning the characteristic phase angle (Figure 4b), healthy subjects (11.94 degrees) has higher *θ*_*c*_ than cancer patients (9.96 degrees), in agreements with previous reports [25,29,30]. In terms of sex, the predicted *θ*_*c*_ values for both female and male participants are also highly consistent with the experimental data, indicating robust performance regardless of gender. Larger characteristic phase angle is observed in male subjects, in line with previous works [25,29,30,31]. The quartile categories show a similar level of accuracy. Lower *θ*_*c*_ values are found in subject located at quartile 3 and 4, where the majority of the cancer patients are located. For centile categories (50, 75, 95 and 100%), the effectiveness in predicting phase angles across different percentiles is demonstrated, subjects with lowest *θ*_*c*_ are located at 100% and the higher at 50% centiles. The BIVA status categories highlights the model's ability to predict *θ*_*c*_ across different BIVA statuses. In addition, from 31 to 44 BIVA statuses the characteristic phase angle decrease gradually, reaching its lower value at 44.

From Figure 4c, again, lower characteristic reactance is reasonably found for cancer patients, in accordance with cell membrane deterioration reported [25,26,27,28,29,30,31]. By sex, female subjects having higher Xcc indicates better cell membrane integrity and overall well-being [25,26,27,28,29,30]. Quartiles 3 and 4 have lower Xcc, where the majority of the cancer patients are located. Higher Xcc is encountered for subjects located at 50% centile, while 75% and 95% have similar values and at 100% centile has the lowest Xcc, at 50–100% centiles the cancer patients are located. Similar behaviour is found for the BIVA statuses. From Figure 4d, the characteristic resistance in cancer patients is slightly higher compared to that of the healthy individuals, which can be attributed to unbalanced body composition in cancer patients. Notably, lower Rc values are observed in male individuals, those in the third quartile, and within the 75–95% centile range. Additionally, Rc exhibits variations across different BIVA statuses.

**Fig. 5:**
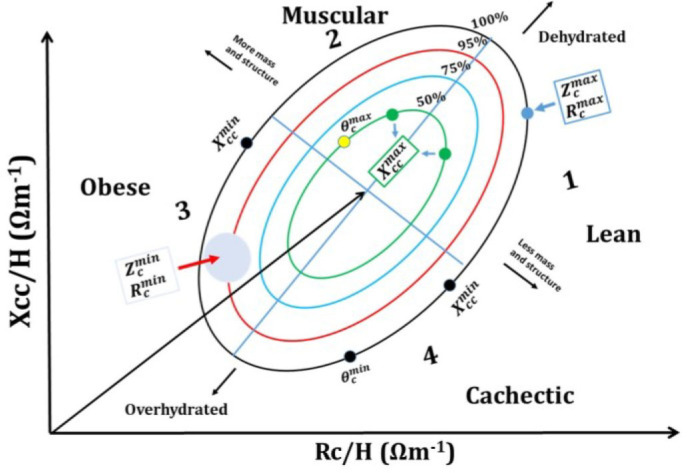
Schematic representation of BIA vector analysis (BIVA), presenting the maximum and minimum values of Zc, *θ*_*c*_, Xcc and Rc (relative to their respective medians).

In order to evaluate the model's performance, a schematic representation of BIVA is presented in Figure 5. The maximum and minimum values of Zc, *θ*_*c*_, Xcc and Rc (relative to their respective medians) are located on the tolerance ellipses. As shown in the figure, the model identifies lean individuals with maximum $\text{Zc}\left(Z_{c}^{\max}\right)$ and $\text{Rc}\left(R_{c}^{\max}\right)$ at quartile 1, centile 100%, indicating less cellular mass and structure. Additionally, athletic individuals with maximum $\theta_{c}\left(\theta_{c}^{\max}\right)$ and $\text{Xcc}\left(X_{\mathrm{cc}}^{\max}\right)$ are located at quartile 2, centile 50%, reflecting balanced hydration and cellular mass. The model predicts that obese subjects have minimum $\text{Zc}\left(Z_{c}^{\min}\right)$ , $\text{Rc}\left(R_{c}^{\min}\right)$ and $\text{Xcc}\left(X_{\mathrm{cc}}^{\min}\right)$ located at quartile 3 between the 75th and 95th percentiles. Meanwhile, cachectic subjects are located at quartile 4, centile 100%, with lower $\theta_{c}\left(\theta_{c}^{\min}\right)$ and $\text{Xcc}\left(X_{\mathrm{cc}}^{\min}\right)$ .

Previous studies have reported that individuals with pathologies associated with hydration status are located in quartile 1, while athletes or people who exercise regularly are in quartile 2. The model can also assign the location of healthy individuals between quartiles 1 and 2, centile 50%. Additionally, obese subjects, hemodialysis patients, and individuals with chronic renal failure undergoing hemodialysis are found in quartile 3. Patients diagnosed with cancer, anorexia nervosa, human immunodeficiency virus (in various stages), COVID-19, etc., are located in quartile 4 [2,29]. Our predictive model aligns with these previous studies. Thus, the model developed in this work is significant for assigning locations without the need for BIVA methods, enhancing clinical assessments and health monitoring.

## Conclusion

In this work, a predictive classification and regression learner models is used to study the association between bioparameters at the characteristic frequency with the location at the tolerance elipses of a cohort from the southern Cuban region. We used 16 characteristics (features) derived from bioimpedance measurements, including other physical parameters. The classification model shows that the bioparameters derived at the characteristic frequency play a crucial role determining the health status and the location parameters. From the regression models, we found a strong agreement between experimental and predicted values for Zc, *θ*_*c*_, Xcc and Rc (resistance) across various categories, including health status, sex, quartile, centile, and BIVA status. The model accuracy is proved, with minimal deviation between predicted and experimental values, indicating its effectiveness in capturing these characteristic electrical parameters. Cancer patients exhibit higher Zc and slightly lower *θ*_*c*_ and Xcc values due to unbalanced body composition and cell membrane deterioration. Females generally show higher Zc and Xcc values, indicating better cell membrane integrity compared to males. The predictions are consistent across quartiles and percentiles, with lower *θ*_*c*_ observed in higher quartiles and centiles where more cancer patients are located. Lastly, variations in Rc values across different BIVA statuses highlight the robustness in estimating impedance parameters, derived at the characteristic frequency, in diverse physiological conditions. The predictive models developed in this study are highly valuable for location assignment without the need to implement bioimpedance vector analysis methods.

While the predictive models presented in this study demonstrate strong agreement between experimental and predicted values, certain limitations must be acknowledged. The dataset is specific to a cohort from the southern Cuban region, which may limit the generalizability of the findings to other populations with different physiological or environmental conditions. For future directions, expanding the dataset to include diverse ethnic, geographic, and physiological groups could enhance the robustness of the models across populations.
